# UBE2O promotes the proliferation, EMT and stemness properties of breast cancer cells through the UBE2O/AMPKα2/mTORC1-MYC positive feedback loop

**DOI:** 10.1038/s41419-019-2194-9

**Published:** 2020-01-06

**Authors:** Xu Liu, Fei Ma, Chunxiao Liu, Kaiyuan Zhu, Wenjie Li, Yuting Xu, Ge Li, Zhenbo Niu, Jiaxin Liu, Du Chen, Zhigao Li, Yingqiang Fu, Cheng Qian

**Affiliations:** 1 Department of breast cancer surgery, Harbin Medical University Cancer Hospital, Harbin Medical University, Harbin, Heilongjiang Province China; 2 North China Translational Medicine Research Center of Harbin Medical University, Harbin Medical University, Harbin, Heilongjiang Province China

**Keywords:** Breast cancer, Cell migration, Cell proliferation, Cancer

## Abstract

Ubiquitin-conjugating enzyme E2O (UBE2O) is a large E2 ubiquitin-conjugating enzyme that possesses both E2 and E3 ligase activities. Ectopic UBE2O overexpression is associated with a variety of human diseases, especially cancers. However, the expression profile and functional biology of UBE2O in human breast cancer (BC) remain unclear. In this study, we found that UBE2O was significantly overexpressed in human BC tissues and cells. Patients with high UBE2O expression tended to have a high risk of metastasis and poor prognosis. In vitro assays revealed that UBE2O promoted BC cell proliferation and epithelial–mesenchymal transformation (EMT) and endowed BC cells with cancer stemness properties (CSPs). UBE2O knockdown in MDA-MB-231 cells suppressed tumour growth and lung metastasis in MDA-MB-231 xenograft mouse models. Mechanistically, UBE2O functioned as a ubiquitin enzyme of AMPKα2, promoting its ubiquitination and degradation and thus activating the mTORC1 signal pathway and contributing to BC oncogenesis and metastasis. Furthermore, as a downstream factor of the UBE2O/AMPKα2/mTORC1 axis, the oncoprotein MYC transcriptionally promoted UBE2O and formed a positive feedback loop in human BC. Collectively, our study demonstrated that UBE2O/AMPKα2/mTORC1-MYC forms a positive feedback loop in human BC cells that regulates BC cell proliferation and EMT and endows BC cells with CSPs.

## Introduction

Ubiquitin-conjugating enzyme E2O (UBE2O) is an E2 ubiquitin-conjugation enzyme that acts as a combination of E2 and E3 enzymes and has both E2 and E3 activities^1^. It is generally expressed in mammalian tissues but is present at higher levels in the brain, heart, skeletal muscle and liver^2^. The deregulation of UBE2O is associated with several human diseases. UBE2O mediates SMAD6 ubiquitination during bone morphogenetic protein signalling^3^ and inhibits TRAF6 K63-polyubiquitination, in turn preventing NF-κB signalling activation^4^. It can also control cellular clock function by ubiquitinating the transcription factor BMAL1^5^. Abnormal UBE2O expression occurs in many types of malignant tumours^6–8^. However, the precise role of UBE2O in human breast cancer (BC) remains unclear.

AMP-activated protein kinase (AMPK) is an intracellular inducer of energy and metabolism. It is a heterotrimer that includes a catalytic α subunit, a regulatory β subunit and a γ subunit^9^. AMPK activation can be regulated by the ATP/AMP ratio or phosphorylation^10^, and its major function is to inhibit anabolism and induce catabolism^11^. AMPK is also an important regulator that controls cellular osmotic pressure and the cellular entry of many xenobiotics^12–14^. The ectopic expression of AMPK is often related to a series of human diseases, especially cancers. Previous studies demonstrated that AMPKα2 downregulation or activity reduction exist in human kidney, ovarian and BCs^15,16^. The antitumour functions of AMPK could be summarised as follows: (1) AMPK can regulate the Hippo pathway, which inhibits cell growth;^17^ (2) AMPK is a downstream target of LKB1, a well-known tumour suppressor;^18^ (3) AMPK activation inhibits ACCA, blocks lipogenesis and reduces tumour growth;^19^ and (4) AMPK can phosphorylate the oncostatin TSC2 and the mTORC1 partner Raptor, thus inhibiting the mTOR pathway^20,21^. However, other studies reported that AMPK activation had a protumour function in RAS-transformed fibroblasts and astrocytes and promoted tumour cell survival^22,23^. AMPK can also alleviate cancer cell metabolic stress and apoptosis via mitochondrial pathways^24,25^. Therefore, the role of AMPK in BC needs to be researched further.

In this study, we demonstrated that UBE2O was significantly overexpressed in BC tissues and cells. Patients with high UBE2O expression tended to have a high risk of metastasis and poor prognosis. Functional assays proved that UBE2O promoted BC cell proliferation and epithelial–mesenchymal transformation (EMT) and conferred BC cells with cancer stemness properties (CSPs). Mechanistically, UBE2O acts as a ubiquitin enzyme of AMPKα2, facilitating its ubiquitination and degradation and thus activating the mTORC1 signalling pathway and contributing to BC oncogenesis and metastasis. Pharmacological intervention of UBE2O inhibited its protumour activities in BC cells by recovering AMPKα2, indicating that UBE2O could be a promising target for BC therapy. Furthermore, as a downstream target of the UBE2O/AMPKα2/mTORC1 pathway, MYC transcriptionally promoted UBE2O expression, which indicated that this axis constitutes a positive feedback loop.

## Materials and methods

### BC cells and specimens

MDA-MB-231, MCF-7 and T-47D cells were purchased from the cell bank of the Chinese Academy of Sciences (Shanghai, China). MDA-MB-453, MDA-MB-468, SK-BR-3, Hs578T, BT-549 and MCF-10A cells were purchased from the BeNa Culture Collection (Beijing, China). MDA-MB-231, MDA-MB-453, MDA-MB-468, T-47D, MCF-7, SK-BR-3 and Hs578T cells were cultivated in DMEM (Gibco, USA) with 10% fetal bovine serum (FBS). BT-549 cells were maintained in RPMI 1640 (Gibco, USA) with 10% FBS. MCF-10A cells were cultured with an MEGM kit containing cholera toxin. All cell lines were incubated at 37 °C in a humidified atmosphere containing 5% carbon dioxide and 95% oxygen.

Fresh BC tissues and paired normal tissues were acquired from BC patients (*n* = 50) who underwent surgery at the Harbin Medical University Cancer Hospital between April and September 2016. All tissue specimens were collected after resection and stored at − 80 °C immediately. One hundred formalin-fixed, paraffin-embedded primary BC specimens were also obtained from the Pathology Department of the Harbin Medical University Cancer Hospital from 2012 to 2013. All of the patients above had complete clinicopathological information. Patients who underwent adjuvant chemotherapy, immunotherapy or radiotherapy before surgery and those with recurrent tumours, metastatic disease, bilateral tumours, or other previous tumours were excluded. Our study was approved by the Research Ethics Committee of Harbin Medical University. Informed consent was signed by all patients who participated in this study.

### Real-time quantitative PCR (qRT-PCR)

TRIzol reagent (Catalogue Number 15596018, Invitrogen, China) was used to extract total RNA, and cDNA was synthesised with a Rever Tra Ace qPCR kit (Catalogue Number FSQ-201, TOYOBO, Japan). qRT-PCR was conducted using SYBR Green Real-Time PCR Master Mix (Catalogue Number QPK-201, TOYOBO, Japan) on a CFX96 Touch Detection System (Bio-Rad, USA). The 2^−∆∆Ct^ method was used to quantify gene expression. GAPDH was used as a reference gene. The sequences of the primers were as follows: GAPDH, 5′-GGAGCGAGATCCCTCCAAAAT-3′ (F) and 5′-GGCTGTTGTCATACTTCTCATGG-3′ (R); UBE2O, 5′-GAATCCAAAACCAAGAGCGAAG-3′ (F) and 5′-TCATCTCTGCCTTCTTTTAGCA-3′(R); MYC, 5′-GGCTCCTGGCAAAAGGTCA-3′(F) and 5′-CTGCGTAGTTGTGCTGATGT-3′(R).

### Cell counting kit-8 (CCK-8) and colony formation assays

For the CCK-8 assay, cells (2 × 10^3^ per well) were seeded into each well of 96-well plates and incubated in culture medium. At each pre-set time, 10 µl of CCK-8 solution (Catalogue Number C0038, Beyotime, China) was added to each well containing 90 µl of culture medium. Then, the plates were incubated at 37 °C for 2 h, and absorbance was detected at 570 nm.

For the colony formation assay, the indicated BC cells were seeded into six-well plates (500 cells per well), and the medium was refreshed every 2 days. Two weeks later, the cells were fixed with formalin for 30 min. Crystal violet (Catalogue Number C8470, Solarbio, China) was applied to stain the cells, and photographs were taken with the FluorChem M system (ProteinSimple, USA).

### Wound healing and invasion assays

For the wound healing assay, cells were seeded into six-well plates, and scratch wounds were made with sterile micropipette tips after the cells reached 100% confluence. At each pre-set time, the cells were washed with phosphate-buffered saline, and images were photographed under a microscope. Then, the migration rates of the indicated cells were measured and analysed.

For the invasion assay, the upper chambers of 24-well Transwell plates (Coring, USA) were coated with Matrigel (Catalogue Number 356234, Coring, USA). Cells (1 × 10^5^) were suspended in 200 µl of serum-free medium and seeded into the upper chambers of the Transwell plates. Then, 600 µl of 10% FBS medium was added to the lower chambers, and the cells were incubated at 37 °C for 24 h. Afterwards, the chambers were fixed, and the cells were stained with crystal violet. Then, images were taken, and the cells were counted under a microscope.

### Sphere culture and sphere formation assays

Cells were suspended in cancer stemness medium (1 × 10^3^ cells per ml) consisting of DMEM/F-12 (Catalogue Number 12660012, Gibco, USA), 1 × B27 (Catalogue Number 17504044, Invitrogen, USA), 20 ng/ml epidermal growth factor (Catalogue Number PHG0311, Invitrogen, USA), 20 ng/ml basic fibroblast growth factor (Catalogue Number PHG0263, Invitrogen, USA), and 2 mm l-glutamine (Catalogue Number 25030081, Invitrogen, USA). Then, the cells were seeded into ultra-low attachment plates (Thermo Scientific, USA). The culture medium was replaced every 48 h. Two weeks later, the stem spheres were imaged and counted under a microscope.

### Immunohistochemistry (IHC) and immunofluorescent staining

IHC assays were performed on paraffin-embedded specimens from BC patients and mice with a standard streptavidin-peroxidase complex method. The staining results were evaluated and scored with a standard histochemistry score (H-SCORE) independently by three pathologists.

Immunofluorescent staining assays were performed as previously described^26^. In brief, cells were fixed, permeabilized with 0.5% Triton-100 (Solarbio, China) and blocked with 5% bovine serum albumin. Afterwards, the cells were incubated with the following primary antibodies (Cell Signaling Technology, USA): anti-CDH1 (14472) and anti-vimentin (5741). Then, the cells were incubated with the corresponding secondary antibodies (CST, USA) and 4',6-diamidino-2-phenylindole (DAPI) (Thermo Fisher Scientific, USA). Finally, the staining results were imaged by confocal microscopy.

### Western blot and cycloheximide assays

For the western blot assay, BC cells and tissues were lysed with radioimmunoprecipitation assay (RIPA) (Catalogue Number P0013B, Beyotime, China) buffer containing phenylmethylsulfonyl fluoride (PMSF) (Catalogue Number ST506, Beyotime, China). The lysate was collected, and western blot assays were conducted as previously described^27^. The following antibodies were used for these experiments: UBE2O (15812-1-AP), AMPKα1 (10929-2-AP), AMPKα2 (18167-1-AP), CDH2 (22018-1-AP), CD44 (15675-1-AP), ABCG2 (27286-1-AP), MYC (10828-1-AP) and β-actin (60008-1-Ig), which were purchased from the Proteintech Group. CDH1 (14472), vimentin (5741), Slug (9585), OCT4 (2750), Raptor (2280), PS792-Raptor (2083), S6K (2708) and PT389-S6K (9234) were purchased from CST.

For the cycloheximide (CHX) assay, MDA-MB-231 cells were seeded into six-well plates and cultured overnight. Then, CHX (200 µg per ml) (Catalogue Number C7698-5G, Sigma, USA) was added to the culture medium. The cells were collected at scheduled times (0, 2, 4, 6 and 8 h) and subjected to western blotting.

### Lentiviral transfection

To establish stable UBE2O overexpression cells, UBE2O expression lentiviral vectors and empty vectors (Genechem, China) were transfected into MCF-7 cells with polybrene (8 μg per ml). Twenty-four hours later, the cells were selected using puromycin, and stable UBE2O expression cells were acquired.

To construct stable UBE2O or AMPKα2 knockdown cells, shRNAs targeting UBE2O and AMPKα2 were designed (Sigma, USA). The shRNA sequences were as follows: UBE2O-shRNA#1: 5′-CCGGCGATGATTCCTATGGCTTCTACTCGAGTAGAAGCCATAGGAATCATCGTTTTT-3′; UBE2O-shRNA#2: 5′-CCGGGACATCAAGAAGCTACAGGAACTCGAGTTCCTGTAGCTTCTTGATGTCTTTTT-3′; UBE2O-shRNA#3: 5′-CCGGCGGGTCTCTTCTTCGATGATTCTCGAGAATCATCGAAGAAGAGACCCGTTTTT-3′; AMPKα2-shRNA#1: 5′-CCGGCGCAGTTTAGATGTTGTTGGACTCGAGTCCAACAACATCTAAACTGCGTTTTT-3′; AMPKα2-shRNA#2: 5′-CCGGGTGGCTTATCATCTTATCATTCTCGAGAATGATAAGATGATAAGCCACTTTTT-3′; and AMPKα2-shRNA#3: 5′-CCGGCCCACTGAAACGAGCAACTATCTCGAGATAGTTGCTCGTTTCAGTGGGTTTTT-3′. The efficiency of transfection was evaluated by qRT-PCR and western blotting.

### Plasmids and RNA interference

Flag-tagged UBE2O, MYC-tagged AMPKα2 and His-tagged ubiquitin plasmids were constructed by Genechem (Genechem, China). All constructs were verified by full-length sequencing. siRNAs targeting MYC were purchased from GenePharma (Shanghai, China). Transient transfection was performed with Lipofectamine 2000 transfection reagent (Catalogue Number 11668019, Invitrogen, USA) according to the manufacturer's instructions. The sequences were as follows: MYC-RNAi#1: 5′-GAGGAUAUCUGGAAGAAAUTTAUUUCUUCCAGAUAUCCUCTT-3′; MYC-RNAi#2: 5′-GCUUGUACCUGCAGGAUCUTTAGAUCCUGCAGGUACAAGCTT-3′; and MYC-RNAi#3: 5′-GGAAGAAAUCGAUGUUGUUTTAACAACAUCGAUUUCUUCCTT-3′.

### Immunoprecipitation (IP) and ubiquitination assays

For IP assays, MDA-MB-231 cells were lysed with RIPA buffer containing PMSF, and the samples were separated by centrifugation. Afterwards, some of the lysate was taken for input, and the remainder was incubated with IgG or the appropriate antibodies at 4 °C overnight. Then, beads were added to the mixture, rotated for 4 h and washed with Tris-buffered saline (TBS) containing PMSF. Finally, the samples were boiled and subjected to western blotting. For co-immunoprecipitation (Co-IP) assays, MCF-7 cells were simultaneously transfected with Flag-tagged UBE2O and MYC-tagged AMPKα2 plasmids (GeneChem, China). Forty-eight hours later, the cells were collected and lysed, and the cell lysates were subjected to IP as described above.

The in vitro ubiquitination assays were performed as described in a previous report^28^. In brief, His-tagged ubiquitin plasmids (GeneChem, China) were transfected into the indicated cells and cultured for 48 h. Then, the cells were collected, and the cell lysates were subjected to IP as described above.

### Chromatin immunoprecipitation (ChIP)

A ChIP assay kit (Catalogue Number, P2078, Beyotime, China) was used according to the manufacturer's instructions. In brief, MDA-MB-231 and MCF-7 cells were treated with formaldehyde at a final concentration of 1% to cross-link the target proteins and DNA. Then, a glycine solution was added to terminate the reaction. The samples were separated by centrifugation at 1000 × *g* for 2 min. Then, the cells were resuspended in sodium dodecyl sulphate lysis buffer with PMSF and lysed with an ultrasonic cell disruptor on ice. Afterwards, the DNA was extracted and cleaned using a DNA depuration kit (Catalogue Number D0033, Beyotime, China). Next, the samples were incubated with anti-MYC (CST, USA) or IgG antibodies at 4 °C overnight, and protein A was used to precipitate the compound. Finally, the DNA was purified, and qRT-PCR was performed to detect the promoter fragments of UBE2O. The primers for the UBE2O promoter were 5′-TCCCAGGTTCAAGCGATTTG-3′ (F) and 5′-CATGGCGAAACCCCATCTCTACT-3′ (R).

### Luciferase reporter assay

A double luciferase assay system (Promega, USA) was used according to the manufacturer’s protocol. In brief, wild-type or mutant-type UBE2O promoter luciferase reporter plasmids were transfected into 293 T cells, and different amounts of MYC plasmids were transfected into 293 T cells as well. Forty-eight hours later, the cells were lysed with passive lysis buffer, and luciferase assays were performed. Firefly luciferase activity normalised to Renilla luciferase activity was used as an internal control.

### Animal study

All animal studies were approved by the Medical Experimental Animal Care Commission of Harbin Medical University. For the tumourigenesis assay, six-week-old female BALB/c nude mice (Beijing Vital River Laboratory Animal Technology Co., China) were randomised into two groups (*n* = 6). MDA-MB-231-Luc cells (5 × 10^5^) transfected with sh-UBE2O#1 and sh-NC were injected into the mammary fat pads of mice, respectively. Tumour growth was measured every 5 days. Seven weeks after injection, the mice were imaged using an Xenogen IVIS Spectrum Imaging System (Caliper Life Sciences, USA), and the tumours were collected for IHC. The tumour volumes were evaluated with the following formula: 1/2 (length × width^2^). For the tumour metastasis model, six-week-old female BALB/c nude mice (*n* = 6) were randomised into two groups. MDA-MB-231-Luc cells (5 × 10^5^) transfected with sh-UBE2O#1 and sh-NC were injected into the tail vein of the mice, respectively. Seven weeks after injection, the lungs were imaged and collected for haematoxylin and eosin staining. The sample size of each group was determined according to the previous studies^29–31^.

### Statistical analysis

All the data are presented as the means ± SDs from at least three independent experiments. Statistical analysis was performed using Student’s *t* test or one-way analysis of variance and the variances between the groups which were being statistically compared were similar. For animal studies, no blinding was used. The chi-square test was used to analyse the relationship between UBE2O expression and the clinicopathological features of BC patients. The Kaplan–Meier method and log-rank test were employed to draw survival curves. *P* < 0.05 indicated statistical significance, which was evaluated using SPSS 21.0 software (Statistical Product and Service Solutions, USA).

## Results

### UBE2O was commonly overexpressed in BC tissues and associated with the prognosis of BC patients

**Fig. 1:**
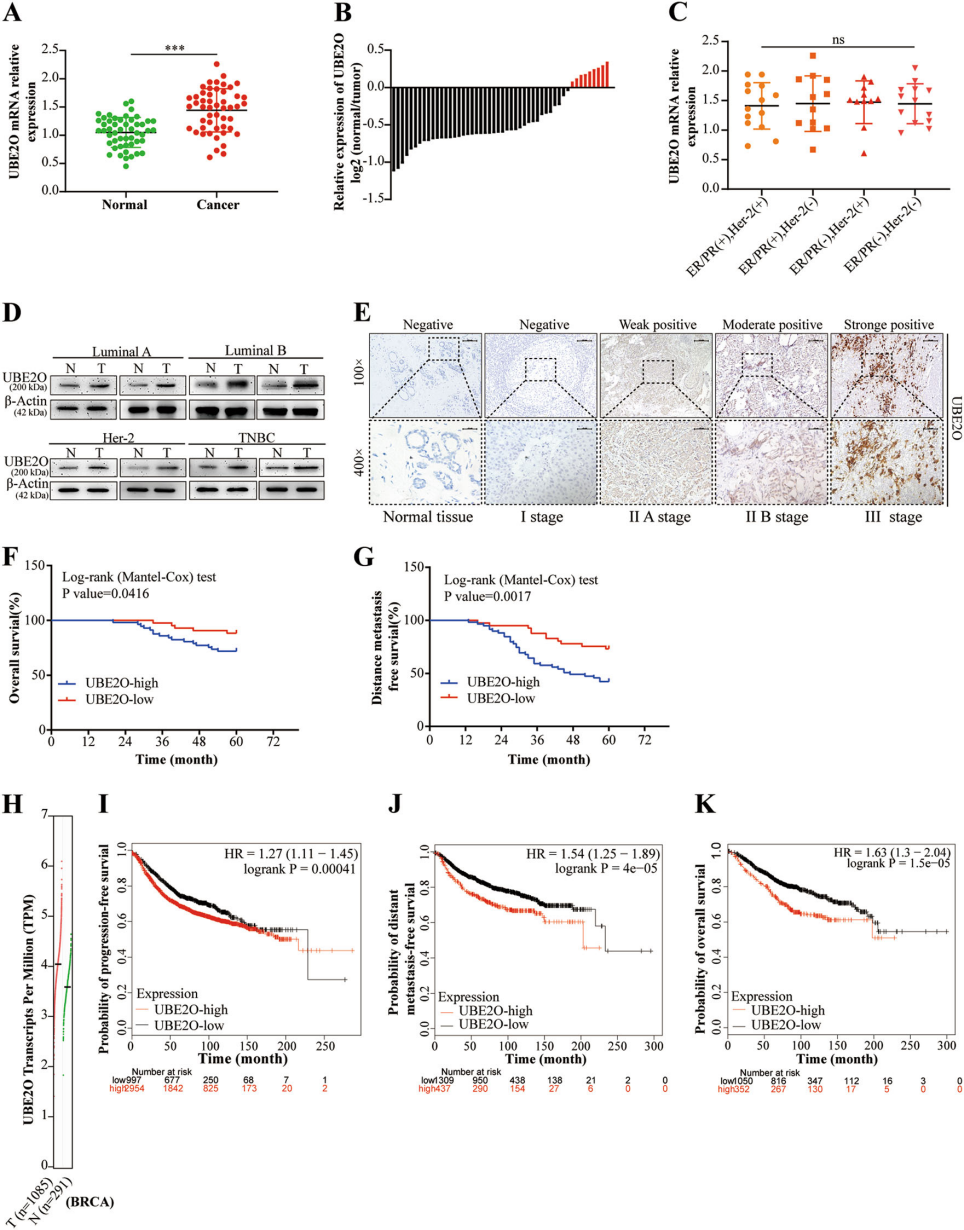
UBE2O was commonly overexpressed in BC tissues and associated with the prognosis of BC patients. **a**, **b** qRT-PCR analysis of UBE2O expression in BC tissues and adjacent normal tissue from BC patients (*n* = 50). **c** qRT-PCR was applied to analyse discrepancies in UBE2O expression in different subtypes of BC tissues (*n* = 50). **d** The protein expression level of UBE2O was analysed in eight cases of different subtypes of BC tissues and corresponding normal tissues by western blotting; β-actin served as a reference. **e** UBE2O expression in different clinical stages of BC tissues and normal mammary tissue was determined by immunohistochemistry (IHC) staining. Representative images were shown (upper: magnification × 100, Scale bar, 100 μm; lower: magnification × 400, Scale bar, 20 μm). **f**, **g** Kaplan–Meier survival curve analysis revealed that patients with high UBE2O expression had poorer overall survival (OS) and distance metastasis-free survival (DMFS) than those with low UBE2O expression. **h** The GEPIA database was used to analyse discrepancies in transcripts from BC patient tissues (*n* = 1085) and normal mammary tissues (*n* = 291). **i–k** Progression-free survival (PFS), DMFS and OS of BC patients with high or low UBE2O expression were analysed by the KMplot database. *p < 0.05, **p < 0.01, ***p < 0.001. The data represent at least three independent experiments.

We sought to detect UBE2O expression in 50 pairs of BC tissues and corresponding normal tissues by qRT-PCR and western blotting. The results showed that UBE2O was significantly upregulated in cancer tissues compared with corresponding normal tissues (Fig. 1a, b). However, no differences in UBE2O expression were found between the four subtypes of BC tissues (Fig. 1c, d). IHC staining was then used to explore the clinical significance of UBE2O expression in BC patients. Representative images of UBE2O expression in different clinical stages of BC tissues and normal breast tissues are shown in Fig. 1e. The clinicopathological features of these patients are shown in Table 1. We found that the UBE2O state was positively correlated with tumour size (*p* = 0.014), axillary lymph node status (*p* = 0.038) and clinical stage (*p* = 0.019). Furthermore, the log-rank test survival analysis confirmed that patients with high UBE2O expression had worse overall survival (OS) (*p* = 0.0416) and distant metastasis-free survival (DMFS) (*p* = 0.0017) (Fig. 1f, g). Given the limited number of patients enrolled in our study, we applied two standard databases to examine the UBE2O status in BC patients. Data from Gene Expression Profiling Interactive Analysis showed that UBE2O transcription was upregulated in tumour tissues (Fig. 1h). Survival information from KMplot indicated that BC patients with high UBE2O expression had worse progression-free survival (PFS), DMFS and OS (Fig. 1i–k). Collectively, these results indicated that UBE2O was highly expressed in BC tissues and negatively correlated with the prognosis of BC patients.

**Table 1:** Association of UBE2O expression and patients’clinicopathological characteristics in invasive ductal carcinoma tissues.

Characteristics	UBE2O expression		No.	*P* value
	High	Low		
*Age*			100	0.589
≤40	4 (50.00%)	4 (50.00%)		
>40	55 (59.78%)	37 (40.22%)		
*ER/PR*			100	
(+)	28 (50.91%)	27 (49.09%)		0.069
(−)	31 (68.89%)	14 (31.11%)		
*HER-2*			100	0.290
0–2 (+)	17 (68.00%)	8 (32.00%)		
3 (+)	42 (56.00%)	33 (44.00%)		
*Tumour size*			100	**0.014**
D ≤ 2 cm	20 (45.45%)	24 (54.55%)		
D > 2 cm	39 (69.64%)	17 (30.36%)		
*Axillary lymph node metastasis*			100	**0.038**
No	31 (50.82%)	30 (49.18%)		
Yes	28 (71.79%)	11 (28.21%)		
*Clinical stages*			100	**0.019**
І–II A	34 (50.75%)	33 (49.25%)		
II B–III	25 (75.76%)	8 (24.24%)		
*Histological grade*			100	0.089
1–2 grade	40 (54.05%)	34 (45.95%)		
3 grade	19 (73.08%)	7 (26.92%)		

Statistically significant difference (*P* < 0.05) are indicated in bold

### Upregulation of UBE2O promoted BC cell proliferation and EMT

**Fig. 2:**
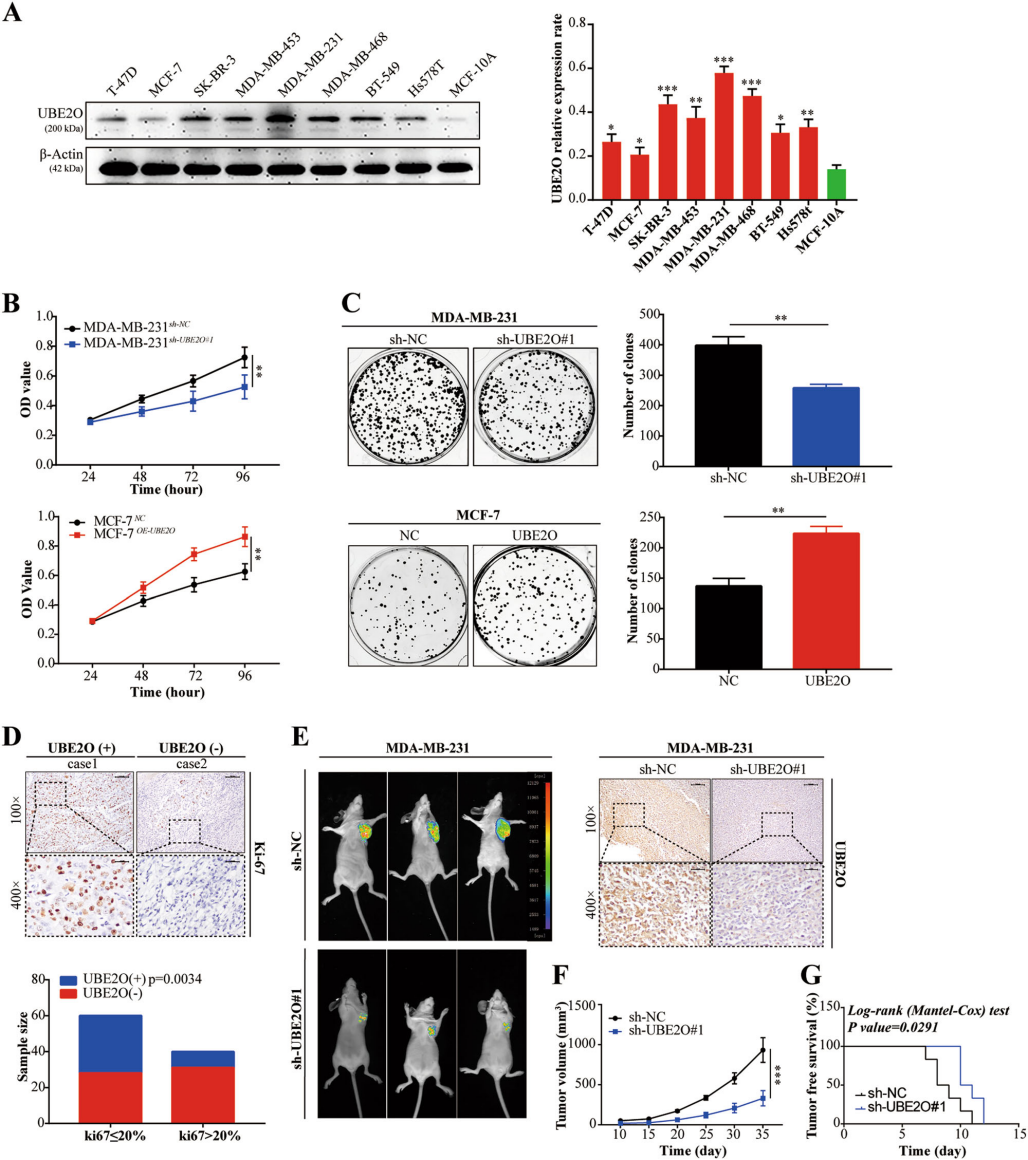
Upregulation of UBE2O promoted BC cell proliferation. **a** Protein expression of UBE2O in a panel of BC cells and nontransformed MCF-10A cells was detected by western blotting. **b** CCK-8 and **c** colony formation assays showed that knocking down UBE2O inhibited the proliferation ability of MDA-MB-231 cells. Conversely, overexpression of UBE2O in MCF-7 cells promoted cell proliferation. **d** The expression status of UBE2O and Ki-67 was examined by IHC (upper: magnification × 100, Scale bar, 100 μm; lower: magnification × 400, Scale bar, 20 μm), and their relevance was analysed by a chi-square test (Ki-67 < 20% was regarded as low expression). **e**–**g** Mouse xenograft models were used to investigate the tumourigenesis of MDA-MB-231^*sh-UBE2O#1*^ cells in vivo (upper: magnification × 100, Scale bar, 100 μm; lower: magnification × 400, Scale bar, 20 μm), **f** the volumes of tumours established in mice in the MDA-MB-231^*sh-NC*^*/*MDA-MB-231^*sh-UBE2O#1*^ groups were recorded, and **g** the tumour-free survival of the two groups was analysed. The data are shown as the mean ± s.d. Student’s *t* test was used for statistical analysis: *p < 0.05, **p < 0.01, ***p < 0.001. The data represent at least three independent experiments.

To explore the function of UBE2O in BC cells, western blot assays were performed with different BC cell lines to examine UBE2O expression. As shown in Fig. 2a, UBE2O was obviously overexpressed in the highly metastatic cell line MDA-MB-231, and the expression was relatively lower in the low metastatic cell line MCF-7. However, the mammary epithelial cell line MCF-10A had the lowest UBE2O expression compared with the other BC cell lines. To directly test the putative cancerogenic function of UBE2O, MDA-MB-231 cells were infected with three specific short hairpin RNAs (shRNAs) with a lentivirus-mediated system to establish UBE2O knockdown cell lines. Meanwhile, MCF-7 was used to establish high UBE2O expression cell lines (MCF-7^*OE-UBE2O*^). The transfection efficiency of the two cell lines was tested by western blotting and qRT-PCR. As shown in Fig. S1a–S1b, sh-UBE2O#1 and sh-UBE2O#3 yielded a relatively better UBE2O knockdown efficiency in MDA-MB-231 cell lines. Thus, MDA-MB-231^*sh-UBE2O#1*^, MDA-MB-231^*sh-UBE2O#3*^ and MCF-7^*OE-UBE2O*^ cells were established and applied for subsequent investigation. Next, we performed CCK-8 assays to detect the effect of UBE2O on BC cell proliferation. The results revealed that UBE2O knockdown reduced the proliferation ability of MDA-MB-231 cells. Conversely, UBE2O overexpression significantly promoted MCF-7 cell growth in vitro (Fig. 2b, Fig. S1c). Colony formation assays also exhibited similar results (Fig. 2c, Fig. S1d). To further explore the correlation between UBE2O status and tumour proliferation in human BC, Ki-67 expression in BC patients was detected by IHC and analysed by chi-square test. The results showed that the UBE2O status was positively associated with Ki-67 expression (Ki-67 > 20% was regarded as a high expression level) in these BC patients (Fig. 2d). Finally, MDA-MB-231^*sh-NC*^ and MDA-MB-231^*sh-UBE2O#1*^cells were injected into the mammary fat pads of BALB/c nude mice. As expected, UBE2O knockdown markedly suppressed xenograft tumour size (Fig. 2e, f) and yielded better tumour-free survival (Fig. 2g). In conclusion, these results confirmed that UBE2O promoted BC cell proliferation both in vitro and in vivo.

**Fig. 3:**
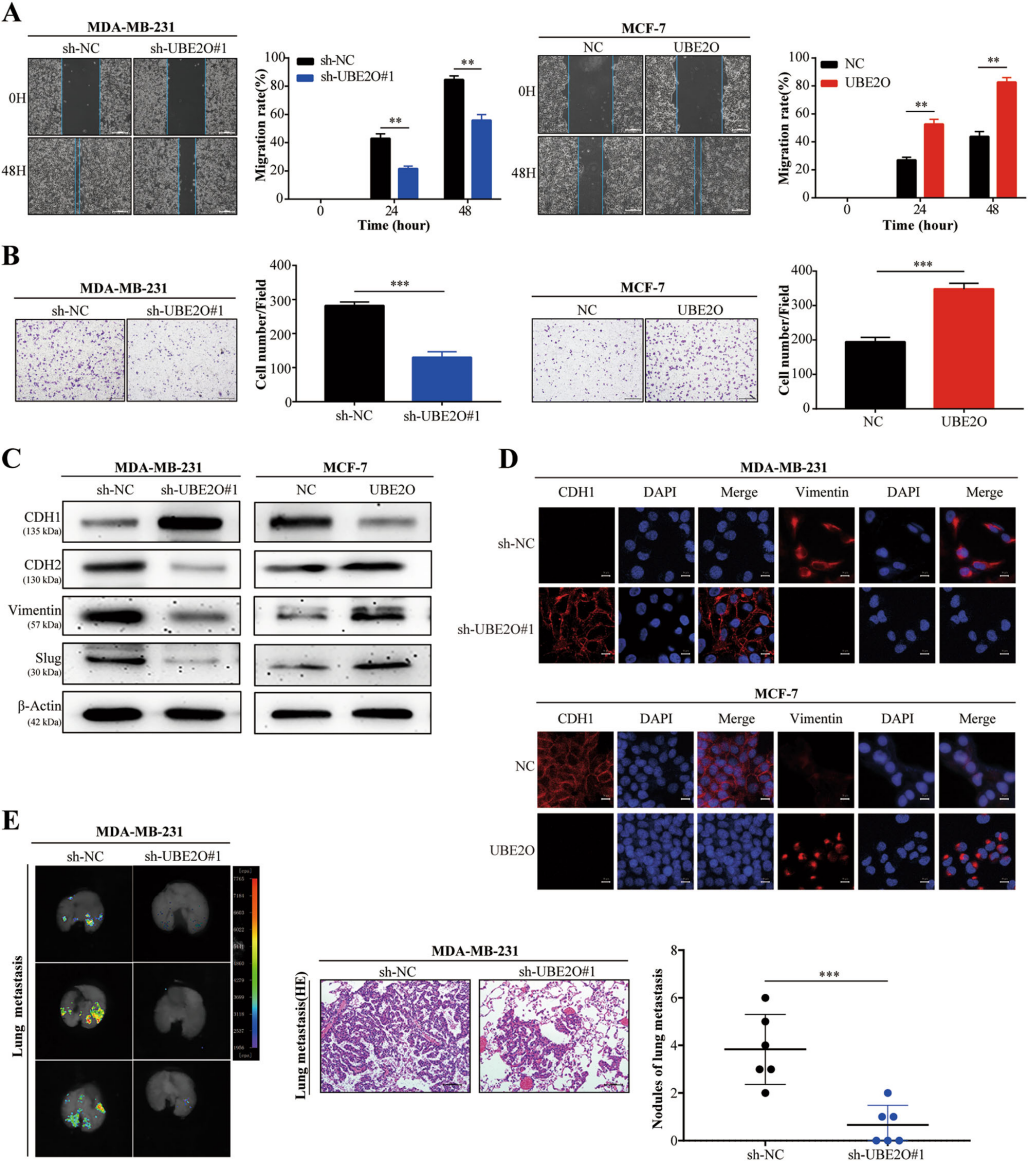
Upregulation of UBE2O promoted BC cell migration and invasion. **a** Wound healing assays were performed to detect the effect of UBE2O expression on migration in the indicated cells (Scale bar, 200 μm). **b** Invasion abilities were examined by Matrigel invasion assays after UBE2O expression levels were changed in the indicated cells (Scale bar, 200 μm). **c** Western blot assays revealed that the epithelial markers (CDH1) were increased, and the mesenchymal markers (CDH2, vimentin and slug) were reduced after inhibiting UBE2O in MDA-MB-231 cells; the opposite results occurred in MCF-7^*OE-UBE2O*^ cells in comparison with control cells. **d** IF staining assays were used to explore EMT markers in MDA-MB-231^*sh-NC*^/MDA-MB-231^*sh-UBE2O#1*^ and MCF-7^*NC*^/MCF-7^*OE-UBE2O*^ cells (Scale bar, 50 μm). **e** Lungs excised from MDA-MB-231^*sh-NC*^/MDA-MB-231^*sh-UBE2O#1*^ mice were photographed, and lung metastases were subjected to HE staining and analysed (Scale bar, 100 μm). The data are shown as the mean ± s.d. Student’s *t* test was used for statistical analysis: *p < 0.05, **p < 0.01, ***p < 0.001. The data represent at least three independent experiments.

Then, we explored the effect of UBE2O on EMT, which is a crucial process in tumour metastasis. In wound healing assays, UBE2O knockdown decreased the migration of MDA-MB-231 cells, and UBE2O overexpression increased the migration capability of MCF-7 cells (Fig. 3a, Fig. S1e). In Matrigel-coated Transwell assays, UBE2O knockdown suppressed the invasive ability of MDA-MB-231 cells, and UBE2O overexpression promoted the invasion of MCF-7 cells (Fig. 3b, Fig. S1f). Furthermore, western blot and immunofluorescence (IF) assays revealed that the epithelial marker CDH1 was upregulated, but the expression of CDH2, vimentin and slug was reduced in MDA-MB-231^*sh-UBE2O#1*^cells. The opposite results occurred in MCF-7^*OE-UBE2O*^ cells (Fig. 3c, d). To investigate the prometastasis effect of UBE2O in vivo, lung metastasis mouse models were established through tail vein injection in another group of nude mice. The results revealed that the mice injected with MDA-MB-231^*sh-UBE2O#1*^cells exhibited fewer lung metastasis nodes than the mice injected with MDA-MB-231^*sh-NC*^ cells (Fig. 3e). In conclusion, these results demonstrated that UBE2O promoted BC cell EMT and metastasis both in vitro and in vivo.

### UBE2O could induce BC cell CSPs

**Fig. 4:**
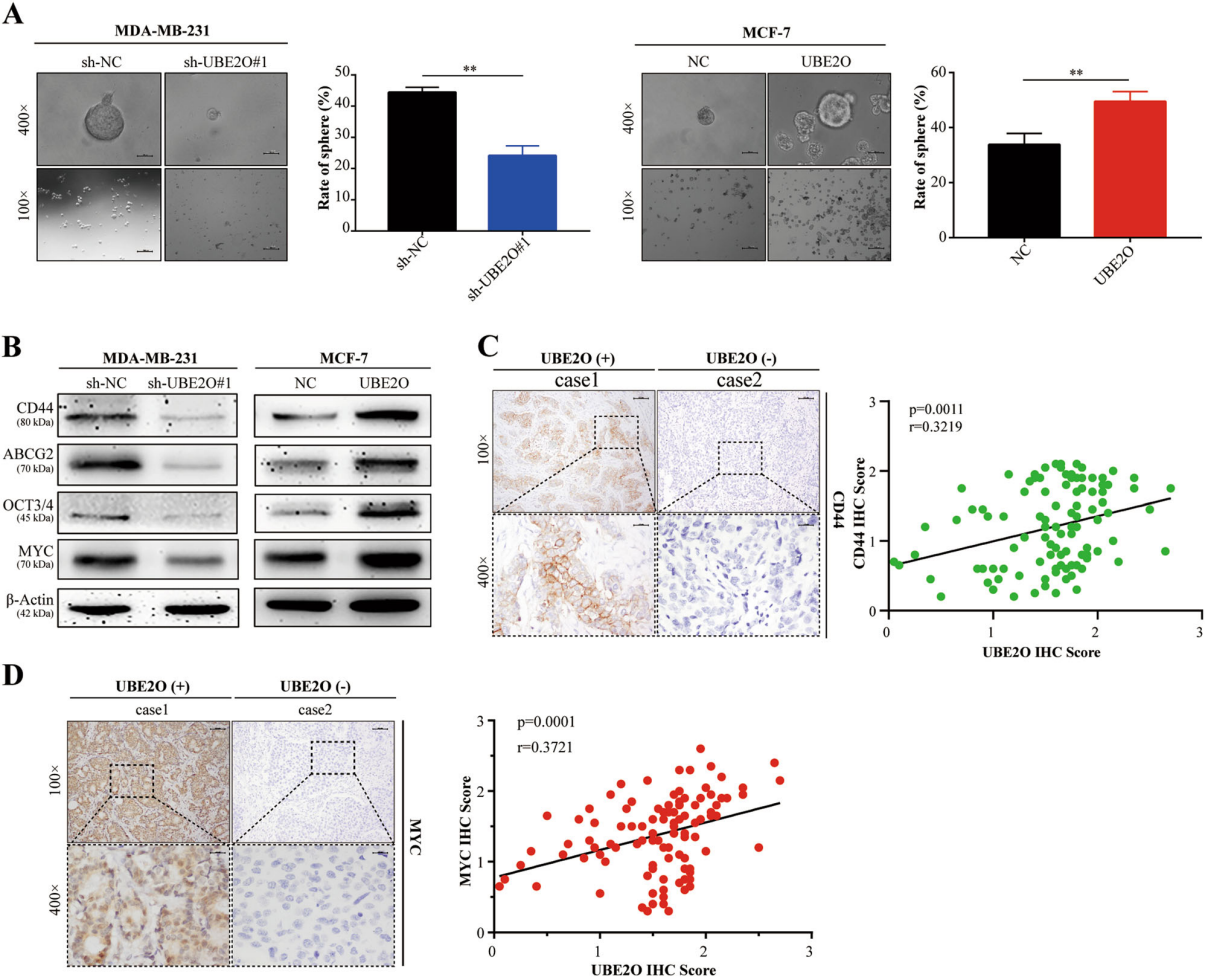
UBE2O-mediated cancer stemness properties in BC cells. **a** The indicated cells were cultured in cancer stemness medium for 2 weeks. Then, the cells were observed under an optical microscope, and the numbers of stem-like spheres formed in each group were determined in triplicate plates (upper: magnification × 100, Scale bar, 100 μm; lower: magnification × 400, Scale bar, 20 μm). **b** Expression levels of cancer stemness markers (CD44, ABCG2, OCT4 and MYC) were determined in MDA-MB-231^*sh-NC*^*/*MDA-MB-231^*sh-UBE2O#1*^ cells and MCF-7^*NC*^/MCF-7^*OE-UBE2O*^ cells by western blotting. **c**, **d** The CD44 and MYC expression status of BC patients were detected by IHC (*n* = 100, upper: magnification × 100, lower: magnification × 400), and their correlation with UBE2O was calculated by Pearson’s correlation coefficient. *p < 0.05, **p < 0.01, ***p < 0.001. The data represent at least three independent experiments.

Given that CSPs play critical roles in BC relapse and metastasis, further studies were carried out to determine whether UBE2O could mediate CSPs in BC cells. Cell stemness sphere assays showed that the sphere formation ability was obviously reduced in MDA-MB-231^*sh-UBE2O#1*^cells, whereas MCF-7^*OE-UBE2O*^cells exhibited greater CSP formation capability (Fig. 4a). Western blot analysis revealed that the CS markers CD44, ABCG2, OCT4 and MYC were significantly reduced in MDA-MB-231^*sh-UBE2O#1*^cells compared with those in the NC group, whereas these markers were increased in MCF-7^*OE-UBE2O*^cells (Fig. 4b). Finally, CD44 and MYC were detected by IHC staining to analyse their correlation with UBE2O expression in clinical samples. Significant positive relationships were found between UBE2O and CD44 or MYC (Fig. 4c, d). Collectively, these results confirmed that UBE2O endowed BC cells with CSPs in vitro.

### UBE2O-mediated AMPKα2 ubiquitination and degradation

**Fig. 5:**
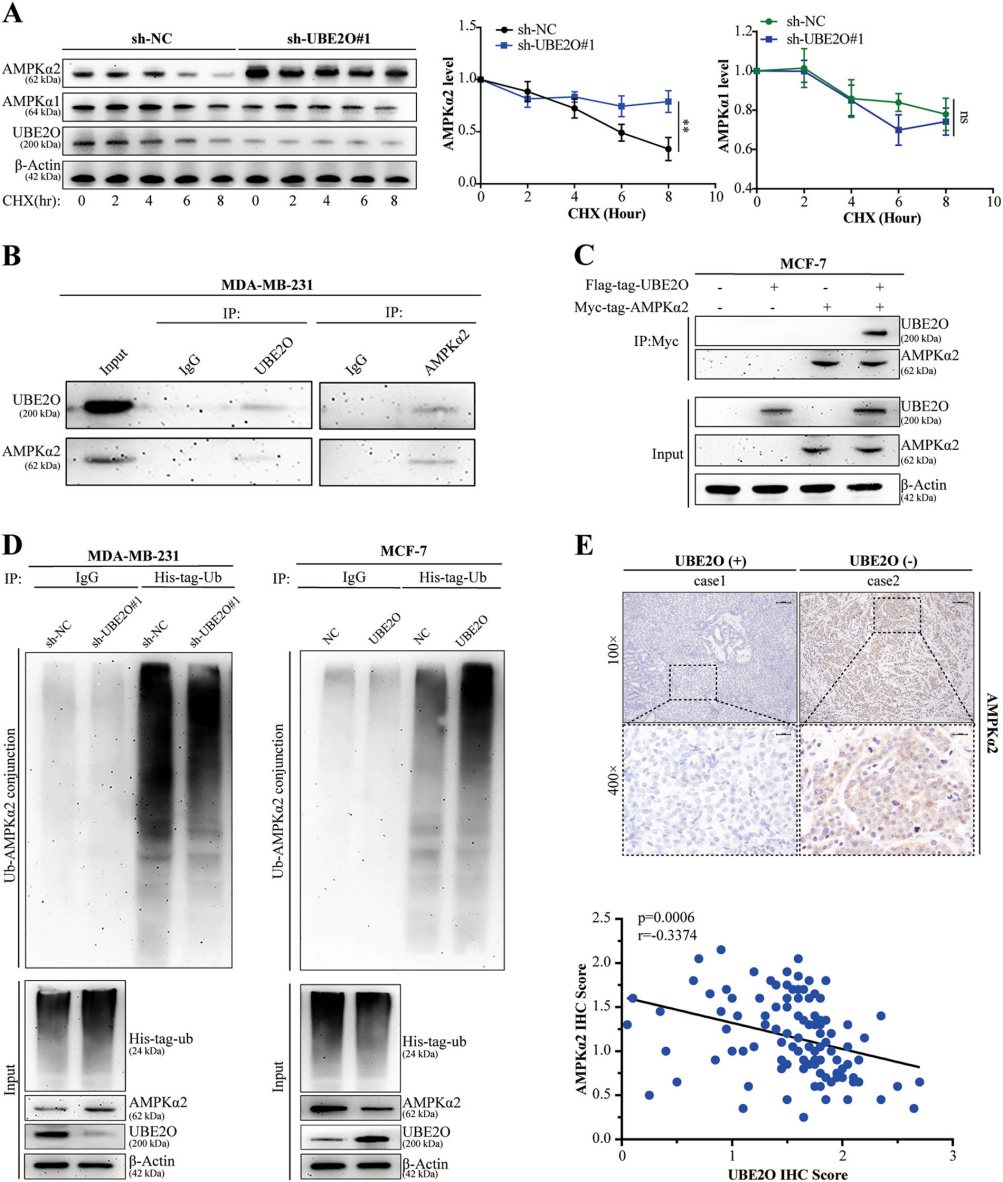
UBE2O-targeted AMPKα2 ubiquitination and degradation. **a** Lysates from MDA-MB-231^*sh-NC*^*/*MDA-MB-231^*sh-UBE2O#1*^ cells treated with cycloheximide (CHX) for the indicated times were subjected to western blotting. AMPKα2 and AMPKα1 expression levels were quantified and normalised to the intensity of the internal reference. **b** Lysates from MDA-MB-231 cells were immunoprecipitated with IgG, anti-UBE2O or anti-AMPKα2 antibodies, and western blot assays were performed. **c** Flag-tagged UBE2O plasmids and MYC-tagged AMPKα2 plasmids were individually transfected or cotransfected into MCF-7 cells. Forty-eight hours after transfection, the cells were lysed, and the lysates were immunoprecipitated with MYC-tag antibodies. Then, western blot assays were performed using anti-UBE2O or anti-AMPKα2 antibodies. **d** Lysates from MDA-MB-231^*sh-NC*^*/*MDA-MB-231^*sh-UBE2O#1*^ cells and MCF-7^*NC*^*/*MCF-7^*UBE2O*^ cells with His-tag ubiquitin were treated with MG132 (10 µm) for 4 h and subjected to IP with IgG or His-tag antibodies and evaluated for ubiquitination with anti-AMPKα2 antibodies. **e** AMPKα2 expression in BC patients was detected by IHC, and its correlation with UBE2O was analysed by Pearson’s correlation coefficient (upper: magnification × 100, Scale bar, 100 μm; lower: magnification × 400, Scale bar, 20 μm). *p < 0.05, **p < 0.01, ***p < 0.001. The data represent at least three independent experiments.

AMPK is a heterotrimer that includes an α subunit (AMPKα), a β subunit (AMPKβ) and a γ subunit (AMPKγ). AMPKα also consists of two structural elements: AMPKα1 and AMPKα2. A previous study demonstrated that UBE2O is an AMPKα2-associated protein that ubiquitinates and degrades AMPKα2 in a series of transgenic mouse models of spontaneous cancer^28^. However, whether UBE2O could ubiquitinate and degrade AMPKα2 in human BC has never been explored. We previously demonstrated that MDA-MB-231 cells had the highest level of UBE2O, so they were used for testing the effect of UBE2O on the stability of AMPKα. CHX assays showed that AMPKα2 protein stability was remarkably enhanced, while AMPKα1 stability was not markedly altered (Fig. 5a). These results confirmed that AMPKα2, but not AMPKα1, was a downstream target of UBE2O and might play a part in UBE2O-dependent tumourigenesis. Next, endogenous IP assays were carried out on MDA-MB-231 cells to further explore the interaction between UBE2O and AMPKα2. Figure 5b showed that UBE2O could combine with AMPKα2 in MDA-MB-231 cells. Co-IP assays also showed that UBE2O could interact with AMPKα2 in MCF-7 cells (Fig. 5c). Then, His-tagged ubiquitin plasmids were transfected into the indicated cell lines, and the intracellular ubiquitination capacity was measured. As shown in Fig. 5d, the ubiquitination of AMPKα2 was significantly reduced in MDA-MB-231^*sh-UBE2O#1*^cells and vice versa in MCF-7 cells. Finally, we used IHC staining to detect AMPKα2 in BC tissues, and a negative correlation between UBE2O expression and AMPKα2 was observed (Fig. 5e). Taken together, these results confirmed that UBE2O could mediate AMPKα2 ubiquitination and degradation in BC cells.

### UBE2O promoted proliferation, EMT and CSPs in BC cells through the UBE2O/AMPKα2/mTORC1 axis

**Fig. 6:**
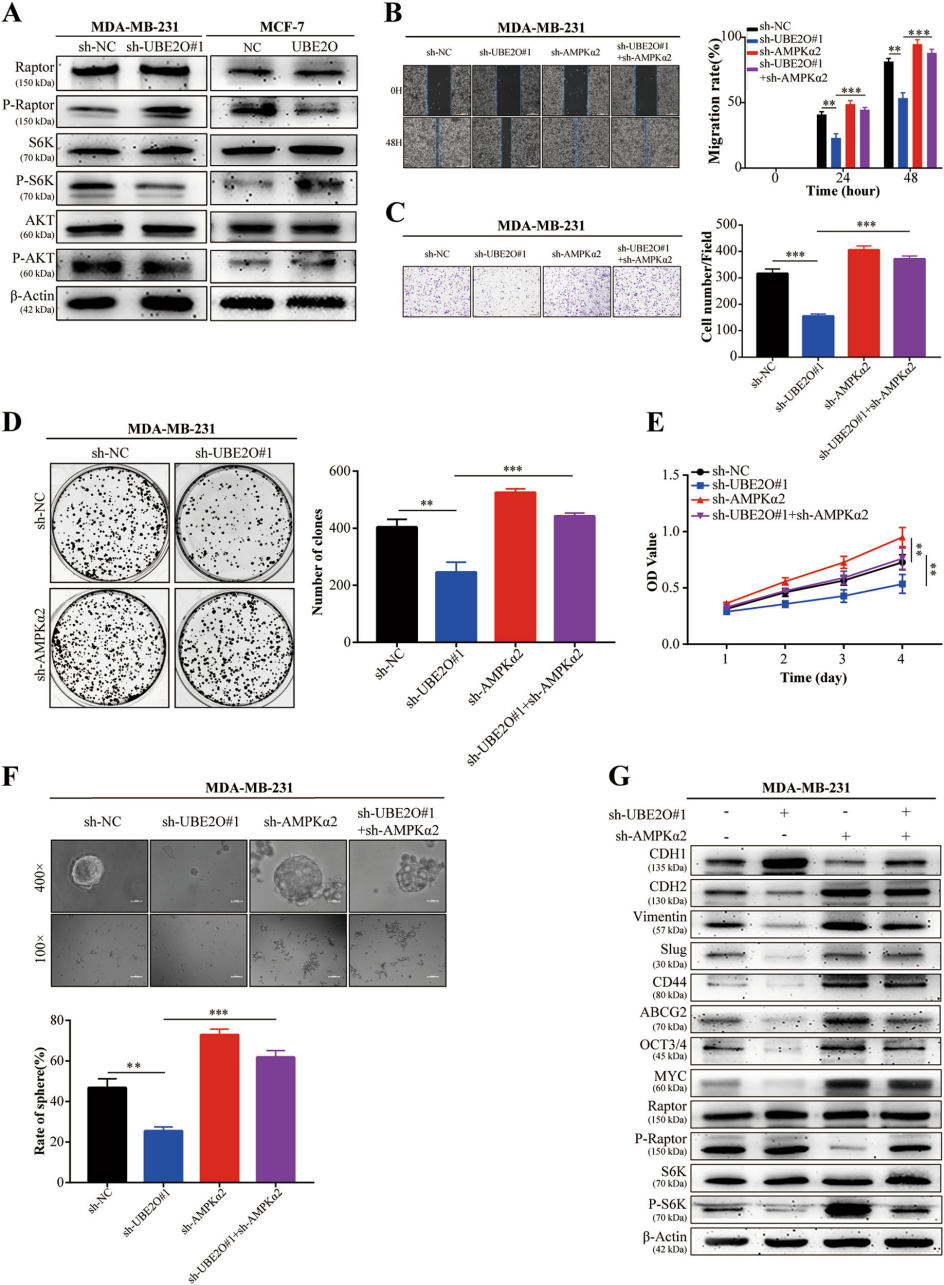
UBE2O induced proliferation, EMT and CSPs in a manner dependent on the regulation of the UBE2O/AMPKα2/mTORC1 axis in BC. **a** Western blotting revealed that p-Raptor expression was increased in MDA-MB-231^*sh-UBE2O#1*^cells, which indicated mTORC1 pathway inhibition. As a downstream effector of mTORC1, p-S6K expression was also decreased. The opposite results occurred in MCF-7^*OE-UBE2O*^ cells. **b**–**f** AMPKα2 was knocked down in MDA-MB-231^*sh-NC*^*/*MDA-MB-231^*sh-UBE2O#1*^cells. Then, **b** scratch assays, **c** invasion assays, **d** colony formation assays, **e** CCK-8 assays and **f** sphere formation assays were performed (upper: magnification × 100, Scale bar, 100 μm; lower: magnification × 400, Scale bar, 20 μm). **g** Western blot assays were performed to detect the expression of EMT markers (CDH1, CDH2, vimentin and slug), CS markers (CD44, ABCG2, OCT3/4 and MYC) and mTORC1 pathway-related markers (p-Raptor and p-S6K) after AMPKα2 suppression in MDA-MB-231^*sh-NC*^*/*MDA-MB-231^*sh-UBE2O#1*^cells. The data are shown as the mean ± s.d. Student’s *t* test was used for statistical analysis: *p < 0.05, **p < 0.01, ***p < 0.001. The data represent at least three independent experiments.

**Fig. 7:**
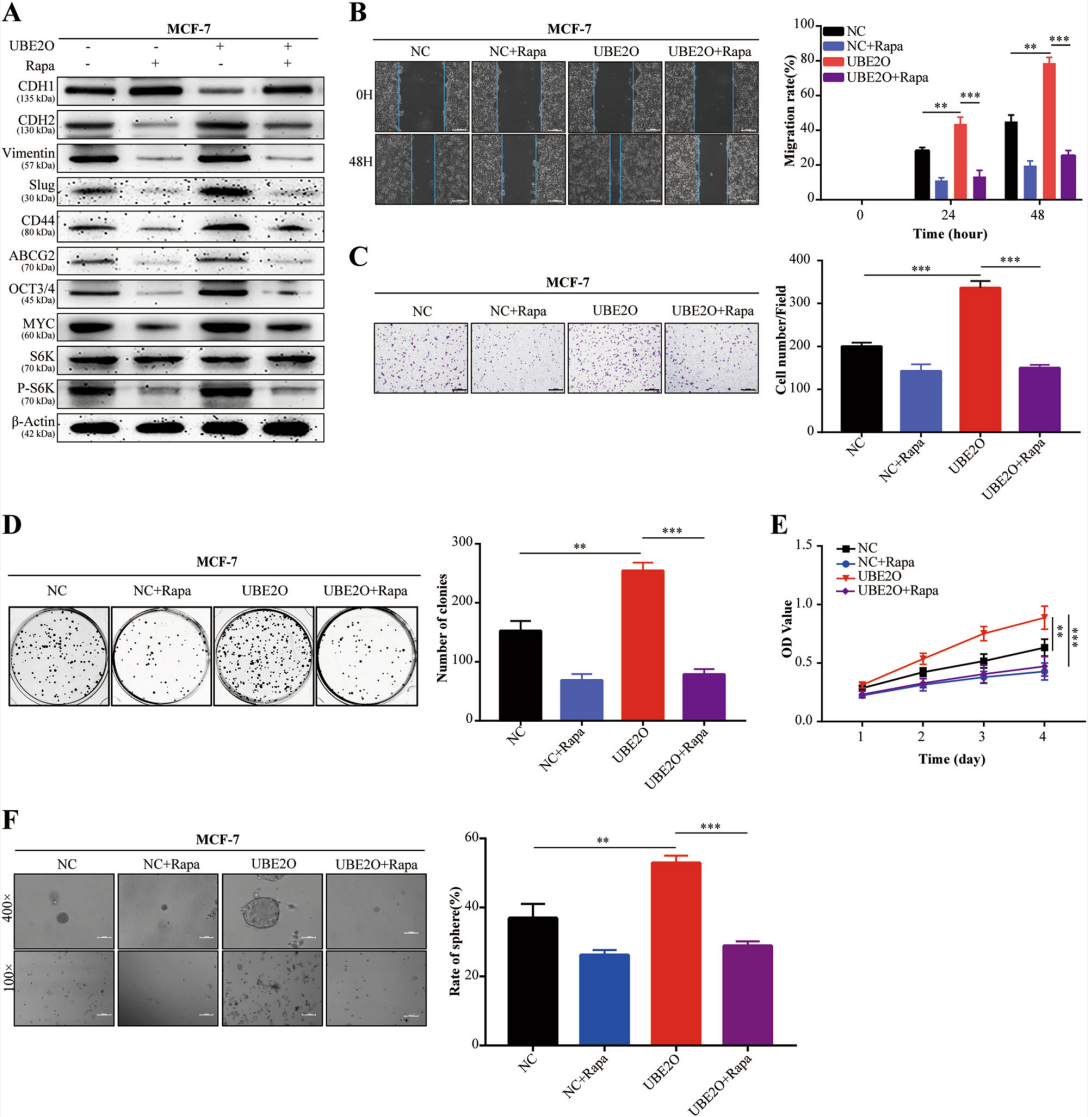
UBE2O induced proliferation, EMT and CSPs in a manner dependent on the regulation of the UBE2O/AMPKα2/mTORC1 axis in BC. **a** MCF-7^*NC*^/MCF-7^*OE-UBE2O*^ cells were treated with rapamycin (100 nm) for 24 h. Then, the cells were lysed, and the expression of EMT markers (CDH1, CDH2, vimentin and slug), CS markers (CD44, ABCG2, OCT3/4 and MYC) and mTORC1 pathway correlative markers was detected by western blot assays. **b**–**f** MCF-7^*NC*^/MCF-7^*OE-UBE2O*^ cells were treated with rapamycin (100 nm), and **b** cell migration, **c** invasion, **d**, **e** proliferation and **f** CSPs (upper: magnification × 100, Scale bar, 100 μm; lower: magnification × 400, Scale bar, 20 μm) were detected. The data are shown as the mean ± s.d. Student’s *t* test was used for statistical analysis: *p < 0.05, **p < 0.01, ***p < 0.001. The data represent at least three independent experiments.

Next, AMPK substrates were detected in the two indicated BC cells, and Fig. 6a showed the enhanced expression of p-Raptor and the decreased expression of p-S6K in MDA-MB-231^*sh-UBE2O#1*^cells, which indicated mTORC1 signalling inactivation. The opposite effects could be observed in MCF-7^*OE-UBE2O*^ cells. Previous studies have used many approaches to show that AMPK has tumour-suppressing functions. Among these, mTORC1 may be the most critical downstream target. AMPK could directly phosphorylate Raptor, the mTORC1-binding partner, and exert its antitumour effect by inactivating mTORC1^20,21^. Our results were in accordance with these studies. However, the expression of another critical upstream regulator of the mTORC1 pathway, p-AKT, failed to show any alterations, which indicated that AMPK, but not AKT, mediated the function of UBE2O in BC. Next, AMPKα2 was stably knocked down in MDA-MB-231 cells (Fig. S2a–S2b), and the phenotypes of the BC cells were analysed. The results revealed that AMPKα2 knockdown could significantly reduce migration (Fig. 6b, Fig. S2c), invasion (Fig. 6c, Fig. S2d), proliferation (Fig. 6d, e, Fig. S2e–S2f) and CSPs (Fig. 6f) in MDA-MB-231 cells. Moreover, western blot assays confirmed that AMPKα2 knockdown also abolished the upregulation of an epithelial marker (CDH1) and the downregulation of mesenchymal markers (CDH2, vimentin) and CS markers (CD44, ABCG2, OCT4 and MYC) in MDA-MB-231^*sh-UBE2O#1*^cells (Fig. 6g). To further explore whether mTORC1 pathway activation was critical for the oncogenic activity of UBE2O in BC cells, MCF-7^*NC*^/MCF-7^*OE-UBE2O*^ cells were treated with rapamycin, an endogenous mTOR inhibitor. Western blot assays showed that the decreased CDH1 expression and enhanced mesenchymal (CDH2 and vimentin) and cancer stemness (CD44, ABCG2, OCT4 and MYC) marker expression were abolished after treating MCF-7^*OE-UBE2O*^ cells with rapamycin (Fig. 7a). Cellular functional experiments verified that the enhanced migration, invasion, proliferation and CSPs of MCF-7^*OE-UBE2O*^ cells were eliminated after treatment with rapamycin (Fig. 7b–f).

Given the crucial roles of UBE2O in BC, chemotherapy strategies targeting UBE2O might be a promising anticancer therapy. A previous investigation demonstrated that AMPK ubiquitination by UBE2O occurred via an intramolecular thioester mechanism. Arsenites can cross-link adjacent cysteines and, in turn, inhibit their ubiquitination^1^. For this reason, a clinically relevant concentration of arsenic trioxide (ATO) (0.5–1 mm), which has been used for treating acute promyelocytic leukaemia, was adopted to inhibit UBE2O ubiquitination. The results in Fig. S3a showed that AMPKα2 expression was remarkably enhanced in MCF-7 cells treated with ATO at clinically relevant concentrations. This result showed that ATO could diminish the polyubiquitination degradation activity of UBE2O toward AMPKα2. Then, we treated MCF-7^*NC*^/MCF-7^*OE-UBE2O*^ cells with ATO and performed cellular functional experiments. Western blot assays confirmed that the upregulation of EMT and cancer stemness markers in MCF-7^*OE-UBE2O*^ cells was blocked by ATO treatment (Fig. S3b). The functional experiments further showed that the enhanced migration, invasion, proliferation abilities and CSPs of MCF-7^*OE-UBE2O*^ cells were abolished by ATO treatment (Fig. S3c–S3f). Collectively, these results showed that UBE2O promoted BC cell proliferation, EMT and CSPs through the AMPKα2/mTORC1 axis and that targeting UBE2O may be a promising strategy for BC therapy.

### MYC transcriptionally promoted UBE2O expression and exerted a positive feedback loop in BC cells

**Fig. 8:**
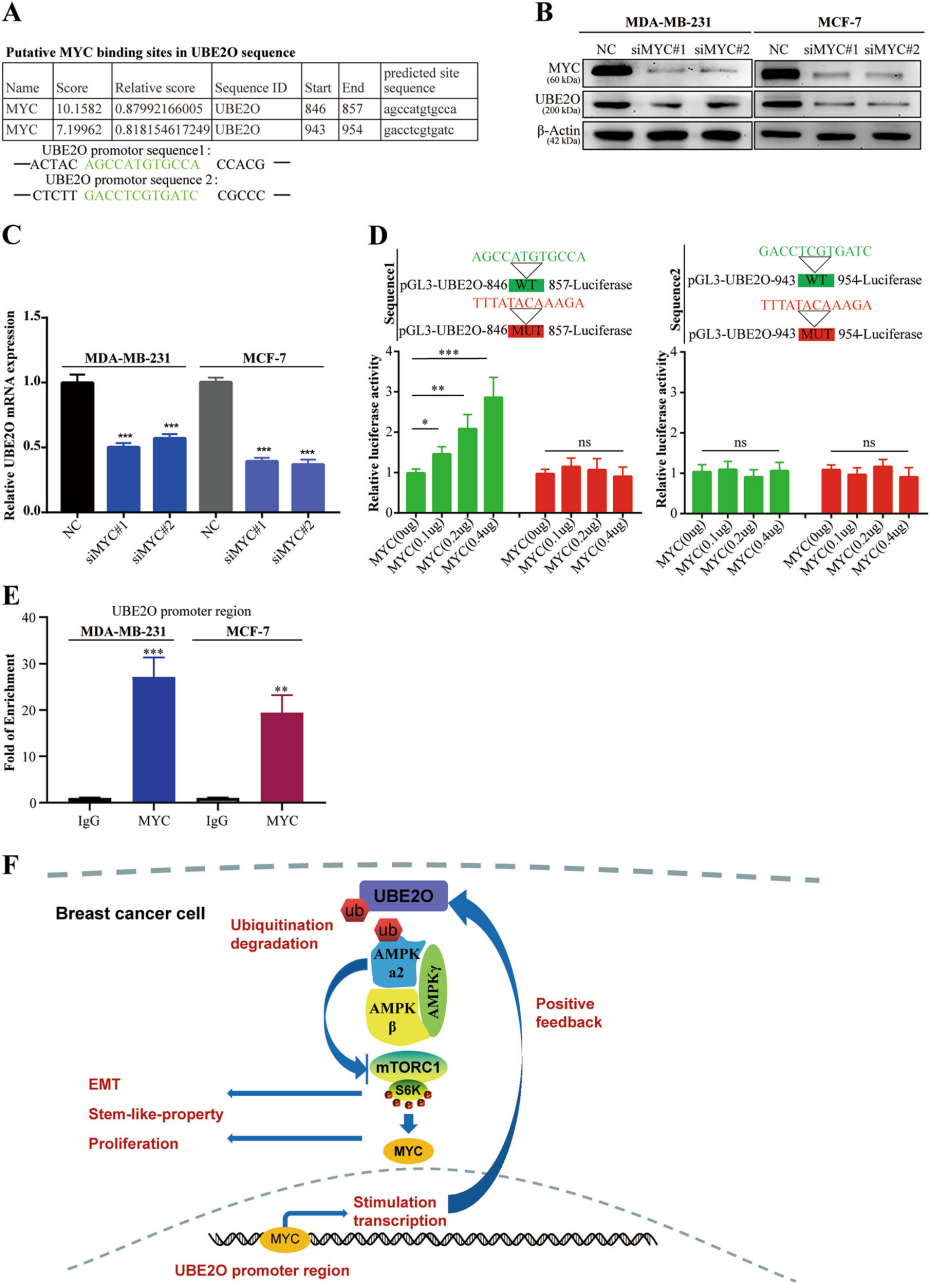
MYC transcriptionally promoted UBE2O expression, and a positive feedback loop existed in BC cells. **a** Two putative MYC-binding sites in the UBE2O promoter region were forecasted by the JASPAR database. **b**, **c** siRNAs targeting MYC were transfected into MDA-MB-231 and MCF-7 cells. Expression changes in UBE2O were detected by qRT-PCR and western blot assays. **d** Wild-type or mutant UBE2O promoter reporter constructs targeting the two putative binding sites of MYC were separately transfected into the 293 T cell line. Various amounts of MYC plasmids were also transfected, and luciferase assays were performed. **e** ChIP assays were performed in the indicated BC cells with IgG or MYC antibodies. qRT-PCR was used to detect the fragments of the UBE2O promoter region. **f** Structure chart showing that the UBE2O/AMPKα2/mTORC1/MYC axis constituted a positive feedback loop in BC cells. The data are shown as the mean ± s.d. Student’s *t* test was used for statistical analysis: *p < 0.05, **p < 0.01, ***p < 0.001. The data represent at least three independent experiments.

As mentioned above, UBE2O was commonly overexpressed in BC, so we investigated the regulatory mechanism of UBE2O in BC cells. The JASPAR database was used to identify potential UBE2O transcription factors. Coincidentally, we found that there were two potential binding sites for MYC in the UBE2O promoter region (Fig. 8a). In addition, we demonstrated that there was a significant linear correlation between UBE2O and MYC in BC patients (Fig. 4d). Therefore, siRNAs targeting MYC were transfected into MDA-MB-231 and MCF-7 cells. The efficiency of MYC silencing was detected by western blotting (Fig. 8b) and qRT-PCR (Fig. S3g), as well as the relative UBE2O expression. The results revealed that UBE2O mRNA and protein expression levels (Fig. 8b, c) were decreased after silencing MYC in MDA-MB-231 and MCF-7 cells. Then, we constructed two sets of luciferase reporter plasmids (sequence 1 and sequence 2) containing the wild-type or mutant MYC-binding sites of the UBE2O promoter region. The data in Fig. 8d revealed that the co-transfection of MYC and UBE2O-sequence 1 reporter plasmids in 293 T cells led to increased luciferase activity, and the rate of increase was positively correlated with the MYC plasmid dose. However, we failed to observe any differences in luciferase activity between the mutant groups. For sequence 2, the luciferase reporter assays showed that there were no significant differences in luciferase activity in the wild-type and mutant plasmid groups with increasing MYC plasmid doses. These results indicated that MYC could bind sequence 1 in the UBE2O promoter region and activate UBE2O transcription. Moreover, ChIP assays confirmed that MYC could directly bind to sequence 1 of the UBE2O promoter-specific region in both MDA-MB-231 and MCF-7 cells (Fig. 8e). Taken together, these results confirmed that MYC could activate the transcription of UBE2O, thus forming a positive feedback loop in BC cells.

## Discussion

From the experiments above, we can draw the following conclusions: (1) UBE2O is highly expressed in BC tissues, and this high expression is related to poor prognosis in BC patients. (2) UBE2O promotes BC cell proliferation and EMT and maintains BC cell CSPs. (3) UBE2O promotes the malignant phenotype of BC cells by mediating AMPKα2 ubiquitination and targeting the UBE2O/AMPKα2/mTORC1 axis. (4) As a downstream oncoprotein of UBE2O, MYC transcriptionally promotes UBE2O expression and exerts a positive feedback loop in BC cells.

As a large E2 ubiquitin-conjugation enzyme, UBE2O displays both E2 and E3 activities, and UBE2O deregulation is involved in various types of human cancers. UBE2O had been proved to decrease the stability of wild-type mixed lineage leukaemia (MLL) in a polyubiquitination-dependent manner, which may accelerate MLL cell proliferation and result in aggressive leukaemia^32^. Vila et al.^28^ introduced UBE2O deletion into a series of transgenic mouse models of spontaneous cancer and revealed that UBE2O could facilitate tumour progression and metastasis in a UBE2O/AMPKα2/HIF-1a-dependent manner. However, the relationship between the UBE2O status of BC patients and their clinicopathological situation has not been fully reported. Our study demonstrated that UBE2O is overexpressed in BC tissues. BC patients with high UBE2O expression tend to have a high risk of tumour metastasis and poor prognosis. Moreover, we used a clinically relevant concentration of ATO to treat BC cells, and the results confirmed that ATO could significantly diminish the protumour ability of UBE2O. Thus, pharmacologically blocking UBE2O might be a promising target for BC treatment.

AMPK is an essential intracellular sensor of energy and metabolism, and its malfunction is associated with many types of human cancers. Gao et al.^33^ reported that the AMPKα2 subunit is mutated at a frequency of 0.2–10% across all human cancers. AMPKα2 deregulation disrupts the tumour suppression capabilities of AMPK and then induces cancer cell progression. Our study confirmed that UBE2O promotes BC cell EMT and endows BC cell CSPs in an AMPKα2 ubiquitination-dependent manner. EMT plays a crucial role in tumour invasiveness and metastasis. During BC progression, EMT furnishes BC cells with self-renewal capability and drives cancer stemness marker expression, contributing to the therapy resistance, immune escape and metastasis of BC cells^34^. In this study, AMPKα2 knockdown remarkably reverses the antiEMT and antiCSP effects in MDA-MB-231^*sh-UBE2O#1*^cells. Treating MCF-7 cells with rapamycin disrupts the proEMT and prostemness properties established by overexpressing UBE2O. These results confirmed that UBE2O mediates BC cell biotumour behaviours in an AMPKα2/mTORC1-dependent manner. In addition, AMPK could inhibit cancer progression by activating p53, p27 and the antiWarburg effect^35–37^. There may also be other antitumour functions of AMPK that remain to be further investigated.

MYC is a well-characterised oncoprotein that is upregulated in 30–50% of BC patients. MYC contributes to BC cell progression and metastasis and induces CSPs in multiple tumour biological processes^38^. MYC acts as a transcriptional factor that regulates the transcription of over 15% of human genes, such as CCND, CDK4 and E2F1, which are involved in tumour progression^39^. In this study, we demonstrated that UBE2O upregulates MYC expression through the AMPKα2/mTORC1 axis and that MYC could bind to the promoter region of UBE2O, thus transcriptionally promoting UBE2O expression. The self-control positive feedback loop further highlights the significance of the UBE2O/AMPKα2/mTORC1-MYC axis in BC.

In conclusion, our study revealed that UBE2O functions as a potential prognostic marker of BC and that the UBE2O/AMPKα2/mTORC1-MYC axis forms a positive feedback loop in BC cells, which promotes BC cell proliferation and EMT and endows BC cells with CSPs. Our research provides crucial theoretical evidence for the function of UBE2O in BC.

## Supplementary information

Supplemental figure.1

Supplemental figure.2

Supplemental figure.3
