# Supplementary figures and images for: UBE2O promotes the proliferation, EMT and stemness properties of breast cancer cells through the UBE2O/AMPKα2/mTORC1-MYC positive feedback loop
Source: Cell Death Dis. 2020 Jan 6;11(1):10. doi: 10.1038/s41419-019-2194-9 (PMC6944706; doi:10.1038/s41419-019-2194-9)

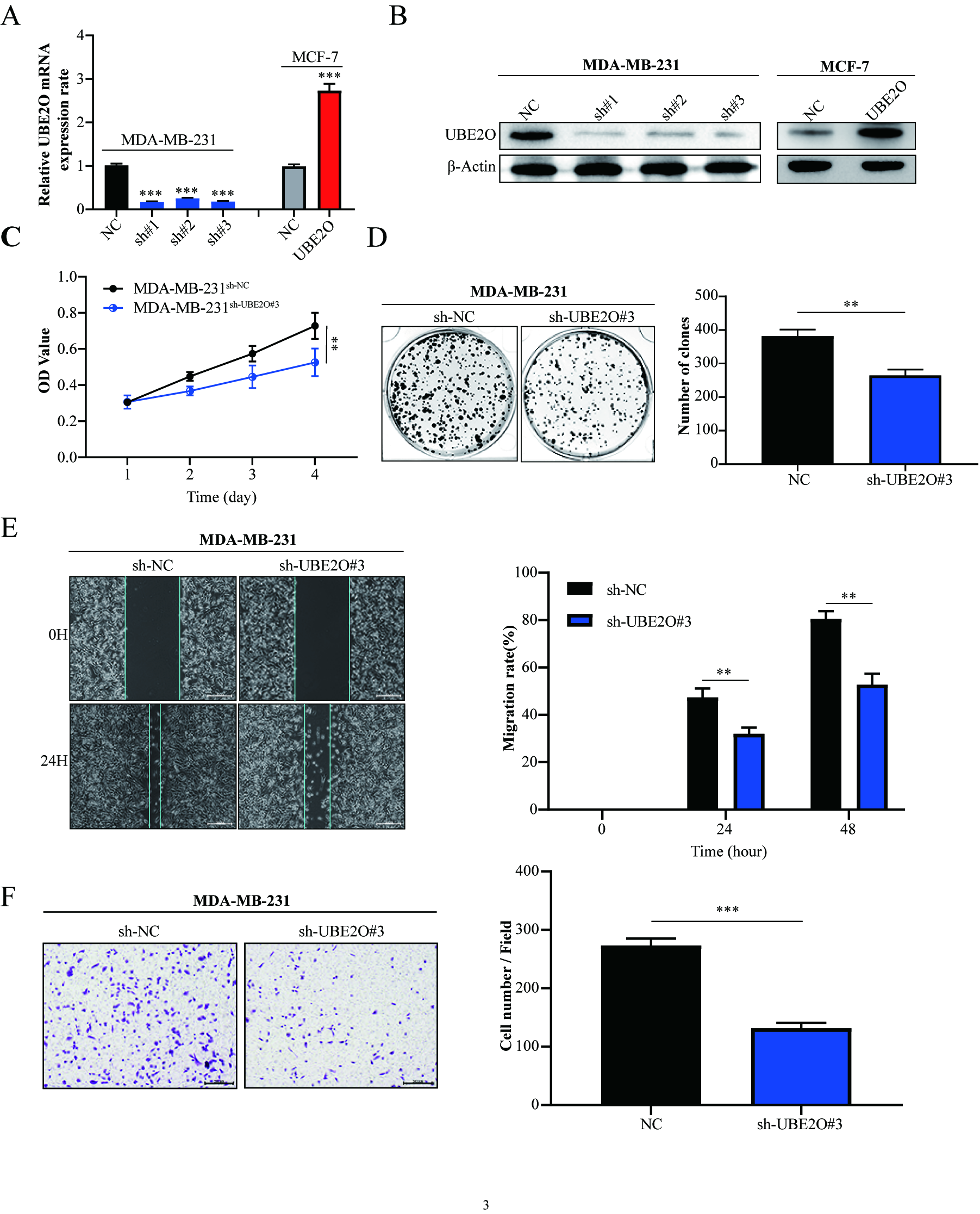

Supplement: Supplementary file 1 — Supplemental figure.1 [file 41419_2019_2194_MOESM1_ESM.tif]

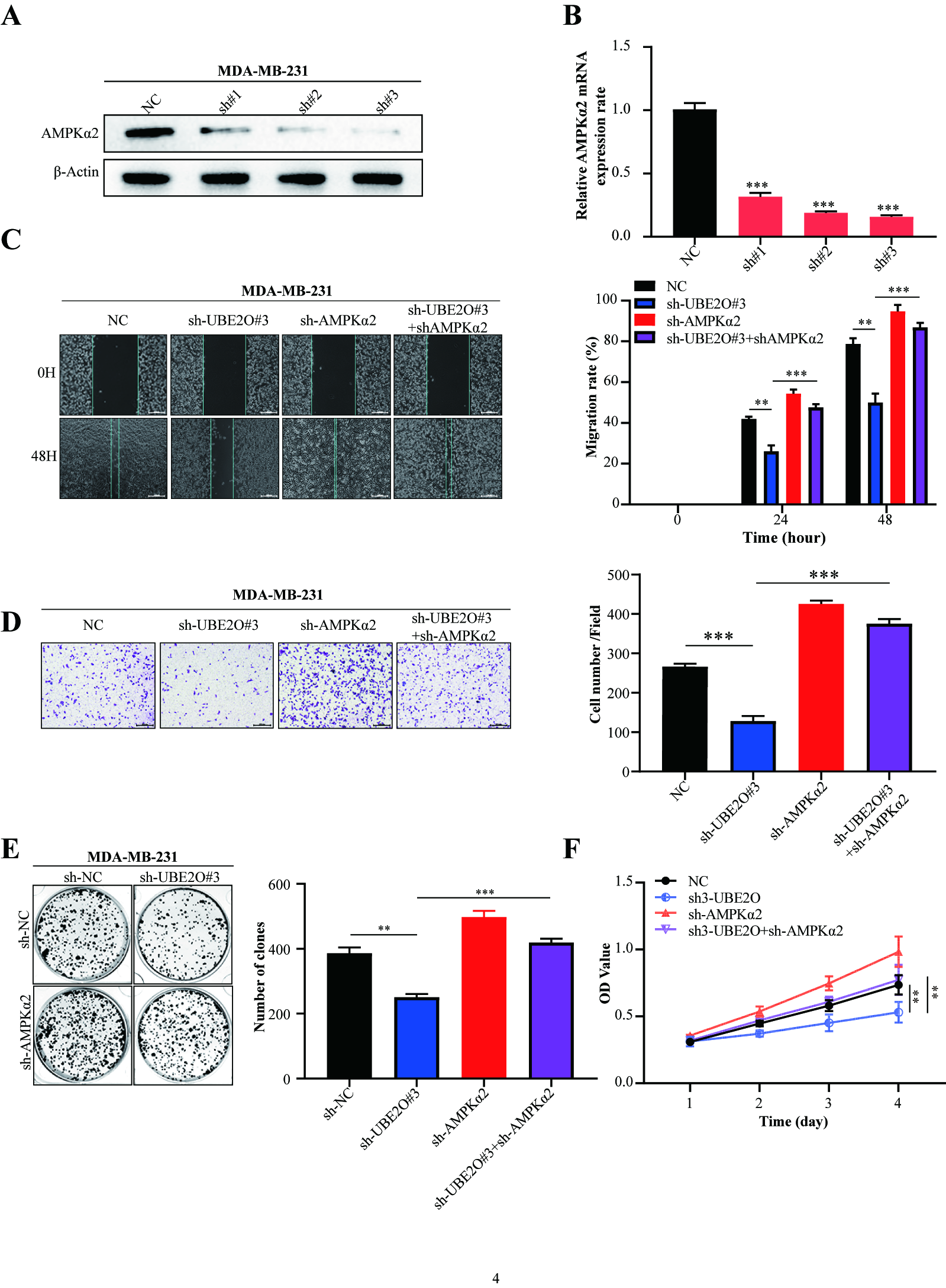

Supplement: Supplementary file 2 — Supplemental figure.2 [file 41419_2019_2194_MOESM2_ESM.tif]

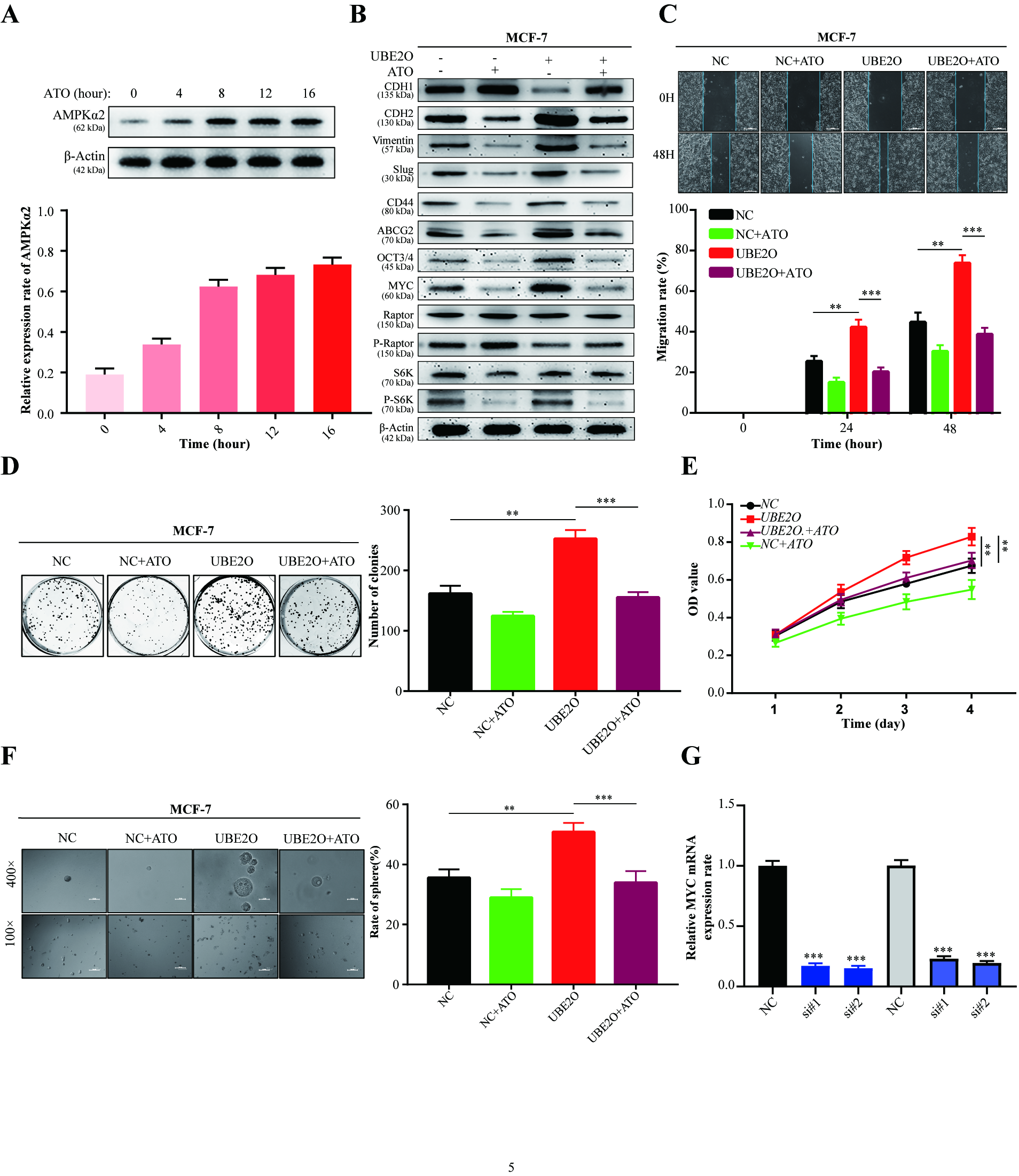

Supplement: Supplementary file 3 — Supplemental figure.3 [file 41419_2019_2194_MOESM3_ESM.tif]
